# Learning parallel and hierarchical mechanisms for edge detection

**DOI:** 10.3389/fnins.2023.1194713

**Published:** 2023-07-25

**Authors:** Ling Zhou, Chuan Lin, Xintao Pang, Hao Yang, Yongcai Pan, Yuwei Zhang

**Affiliations:** ^1^Key Laboratory of AI and Information Processing (Hechi University), Education Department of Guangxi Zhuang Autonomous Region, Hechi University, Yizhou, China; ^2^School of Automation, Guangxi University of Science and Technology, Liuzhou, China; ^3^Guangxi Key Laboratory of Automobile Components and Vehicle Technology, Guangxi University of Science and Technology, Liuzhou, China

**Keywords:** edge detection, convolutional neural network, parallel processing mechanism, hierarchical processing mechanism, lightweight methods

## Abstract

Edge detection is one of the fundamental components of advanced computer vision tasks, and it is essential to preserve computational resources while ensuring a certain level of performance. In this paper, we propose a lightweight edge detection network called the Parallel and Hierarchical Network (PHNet), which draws inspiration from the parallel processing and hierarchical processing mechanisms of visual information in the visual cortex neurons and is implemented *via* a convolutional neural network (CNN). Specifically, we designed an encoding network with parallel and hierarchical processing based on the visual information transmission pathway of the “retina-LGN-V1” and meticulously modeled the receptive fields of the cells involved in the pathway. Empirical evaluation demonstrates that, despite a minimal parameter count of only 0.2 M, the proposed model achieves a remarkable ODS score of 0.781 on the BSDS500 dataset and ODS score of 0.863 on the MBDD dataset. These results underscore the efficacy of the proposed network in attaining superior edge detection performance at a low computational cost. Moreover, we believe that this study, which combines computational vision and biological vision, can provide new insights into edge detection model research.

## 1. Introduction

Edge detection is a fundamental task in the field of computer vision, aimed at extracting clear edge information from complex backgrounds and textures. It plays a crucial role in advanced visual tasks such as object detection (Kyrkou et al., 2013; Dai et al., 2021) and semantic segmentation (Tu et al., 2020; Peng et al., 2021), making it an important area of focus of research in computer vision.

Convolutional Neural Networks (CNN) have been widely used in computer vision due to their excellent feature extraction ability and good performance. In edge detection tasks, CNNs have also shown promising results. In CNN-based edge detection models with an encoding-decoding architecture, a large classification network such as VGG16 (Simonyan and Zisserman, 2014) is typically employed as the encoding network to extract edge feature information. The extracted features from the encoding network are further processed and fused with information through the design of a decoding network to obtain the final edge image. Compared to traditional edge detection methods (Prewitt, 1970; Sobel, 1970; Canny, 1986; Dalal and Triggs, 2005; Wang and Shui, 2016) and bio-inspired visual mechanisms for edge detection (Grigorescu et al., 2003; Petkov and Subramanian, 2007; Yang et al., 2014; Akbarinia and Parraga, 2018), CNN-based edge detection methods can extract most of the feature information in the image, fuse features from different levels, and achieve better performance.

The aforementioned CNN-based edge detection network employs VGG16 as the encoding network, which has the characteristic of having a large number of network parameters, high computational cost, and requiring transfer learning to achieve better edge detection performance. These characteristics are generally present in encoding-decoding edge detection models that rely on transfer learning. This is because these methods usually adopt encoding networks with deeper layer structures, which increase model complexity by stacking a large number of small-sized convolution kernels to increase model depth and width. However, research has shown that a large number of stacked small-sized convolution kernels not only cannot effectively increase the receptive field area as theoretically expected (Luo et al., 2016), but also can cause parameter redundancy (Chen et al., 2016).

Braekevelt (1990) found that compared to human vision, cats are myopic and must be 6 meters away to see what an ordinary person can see at 20 or 30 meters. This means that the edge information obtained by the cat’s visual cortex from the image is not as clear as that of humans, but this does not affect the cat’s ability as a mouse hunter. That is to say, accurate identification of an object does not necessarily require high-quality edge information. From the perspective of “sacrificing a small part of network performance, saving computational cost, and reserving more computing power for other higher-level visual processing tasks,” this article designs a biologically inspired lightweight edge detection network.

This article combines the transmission of biological visual information between different hierarchical levels with convolutional neural networks. Specifically, it corresponds X cells, Y cells, simple cells, and complex cells in the visual pathway to the encoding network. With the aim of achieving fewer parameters, we designed a lightweight biomimetic encoding network that sacrifices some performance while still possessing high practical value. Our contributions can be summarized as follows:

1. Inspired by the principle that visual information is efficiently processed through the parallel and hierarchical mechanisms of visual neurons in the visual cortex, and with the goal of reducing model parameters, this paper proposes a lightweight edge detection deep learning model that simulates the parallel and hierarchical mechanisms of the “retina-LGN-V1” visual pathway.
2. Accurately modeling the receptive field properties of X cells, Y cells, and simple cells involved in parallel and hierarchical processing in the visual cortex, convolution models were built to simulate these properties. Based on these models, a lightweight edge detection network was proposed to simulate the information transmission characteristics and edge detection mechanism in the “retina-LGN-V1” visual pathway.
3. Our network achieves good performance in edge detection tasks with relatively fewer parameters, which leaves more storage and computing resources for subsequent advanced visual tasks.

We have arranged the content of our paper as follows: In Section “Related work”, we introduce the research status of edge detection algorithms from both non-biomimetic and biomimetic perspectives. In Section “Propose method”, we provide the specific details of our proposed PHNet. In Section “Experiment”, we evaluate the performance of PHNet based on the BSDS500 (Arbelaez et al., 2010) and MBDD (Mély et al., 2016) datasets, examining the model’s advantages and disadvantages from three aspects: accuracy, computational complexity, and parameter size. In Section “Conclusions and prospects”, we summarize our research and discuss future research directions based on the results.

## 2. Related work

The task of edge detection has been extensively researched by scholars, resulting in the proposal of numerous edge detection methods. This article classifies the task of edge detection into two broad categories based on the research direction: biologically-inspired edge detection, which draws inspiration from bio-vision, and non-biologically-inspired edge detection, which is designed based on empirical methods.

### 2.1. Biologically-inspired edge detection methods

The biological visual system possesses powerful visual capabilities, allowing for the rapid comprehension of complex natural scenes. Investigating the design of edge detection methods based on the mechanisms by which the visual system processes information is a worthwhile research direction. The concept of receptive fields, as an important property of visual nerve cells, can effectively reflect the response characteristics of these cells to stimuli. Inspired by the findings of Hubel and Wiesel (1962) in the primary visual cortex, Grigorescu et al. (2003) simulated the classical and non-classical receptive fields of primary visual cortex cells using Gabor functions and Gaussian difference functions, respectively. This resulted in the edge detection algorithm acquiring certain texture suppression capabilities. Petkov and Subramanian (2007) proposed Spatiotemporal Gabor filters based on the dynamic characteristics of receptive fields in the primary visual cortex to effectively suppress noise and texture by integrating spatiotemporal information. Yang et al. (2014) simulated the classical receptive field of primary visual cortex cells using Gaussian first-order derivatives and comprehensively considered the features of direction, brightness, and contrast to effectively improve the edge detection performance of the algorithm. Zhong and Wang (2021) explained and modeled the receptive field mechanisms of retinal cells’ light/dark adaptation and simple and complex cells based on the visual information transmission and processing mechanisms from the retina to the V1 area and the visual information degradation mechanism. They proposed an edge detection algorithm that highly mimics the neural mechanism. Akbarinia and Parraga (2018) were inspired by the feedback mechanism of V2 neurons in the higher visual cortex and considered four types of peripheral receptive fields (Full, Far, Iso-, and Orthogonal-orientation) and relied on the contrast adjustment of the four peripheral mechanisms to contribute to the center. They also proposed a feedback connection from higher visual areas to lower visual areas. Fang et al. (2020) proposed an edge detection method that enhances local contrast difference response based on the mechanism of bilateral asymmetric receptive fields in the visual pathway. Meanwhile, the processing of color information by the visual system is essential in visual cognition. Yang et al. (2013) proposed the CO algorithm based on the color opponent mechanism of visual nerve cells’ receptive fields, which simulated red-green and yellow-blue color opposition and can more stably detect color edges in natural images. Subsequently, Yang et al. (2015) added sparse coding for texture suppression in the CO algorithm and proposed the SCO algorithm, which has stronger texture suppression ability than the CO algorithm.

The aforementioned biomimetic edge detection method utilizes a certain function to simulate visual physiological mechanisms. Inspired by the receptive field mechanism of biological vision, Tang et al. (2019) were the first to propose a model that combines CNN with biological vision mechanisms, presenting a multi-scale fusion encoding network that achieved excellent performance. Subsequently, Lin et al. (2022) designed a pre-enhancement network (PENet) by simulating the information transmission mechanism of the retina/lateral geniculate nucleus (LGN), which enhanced the feature extraction capability of the encoding network. They further designed a decoding network that integrated the hierarchical structure of the visual pathway and the integration characteristics of the inferior temporal (IT) cortex, thereby enhancing the feature integration ability of the decoding network.

### 2.2. Non-biomimetic edge detection methods

Early edge detection methods mostly relied on classical mathematical operators. Local brightness gradient detection operators such as Sobel (1970) and Prewitt (1970) were among the earliest edge detection methods, detecting edge information in images by detecting changes in brightness gradient. However, this method is sensitive to noise and texture, and is not robust enough. Wang and Shui (2016) used the first-order partial derivative of Gaussian to perform anisotropic edge detection on color images, proposing the ANND edge detection method. The main component was extracted from the response of different color channels and directions using singular value decomposition to generate an edge intensity map. They also improved the performance by fusing gradient-based and Gaussian first-order derivative-based edge responses. Later, Li and Shui (2021) improved the model’s performance by training a classifier to integrate anisotropic edge responses. Although these methods are constantly being improved and optimized, they still struggle to extract edges well from complex backgrounds, as they lack consideration of different types of features and reference to contextual information, making it difficult to meet the demands of current applications.

In recent years, the development of deep learning technology has led to the proposal of numerous edge detection methods based on deep learning. These methods use convolutional neural networks to map natural images to edge images. Xie and Tu (2017)were inspired by FCN (Long et al., 2015) and DSN (Lee et al., 2015) and proposed an end-to-end HED based on convolutional neural networks. They used the convolutional part of VGG16 as the encoding network, extracted five different scales of features, and used them for edge output prediction after upsampling. Liu et al. (2017)improved HED and proposed the RCF network, which predicts edges using the features of all 13 convolutional layers after upsampling. Wang et al. (2017) addressed the issue of blurry edges caused by direct upsampling to the same resolution in HED and proposed the CED network. They adopted a strategy of slow upsampling and feature fusion at each level to ensure that the multi-scale features in the CED network do not lose accuracy due to excessive upsampling.

However, these edge detection methods require the use of large classification networks such as VGG16 as the encoding network, and rely on transfer learning to achieve high-performance edge detection, which requires a significant amount of computing resources. Therefore, researchers have started to focus on compressing the model’s parameters and computing power while maintaining high performance. At the same time, traditional edge detection algorithms such as Canny (1986), which use pixel relationships, have once again attracted researchers’ attention. Su et al. (2021) combined the gradient information of traditional edge detection operators with CNN and designed a lightweight edge detection network called PiDiNet.

To summarize, edge detection, as the foundation of other advanced visual tasks, should occupy fewer computing resources to reserve more resources for higher-level visual computing tasks. Biological vision has the characteristic of efficiently understanding complex natural scenes. Therefore, this paper proposes a lightweight edge detection model guided by biological vision mechanisms.

## 3. Propose method

### 3.1. Bio-visual concept

Retinal ganglion cells (RGCs) are the most extensively studied and well-characterized cells in the retina. In Kuffler (1953), discovered that they have concentric antagonistic receptive fields, which contain a large number of photoreceptors. The photoreceptors in the center of the receptive field provide excitatory signals to the ganglion cells, while those in the surrounding area provide inhibitory signals. Therefore, researchers divide the receptive fields into excitatory centers and inhibitory surrounds. In Rodieck (1965), established a mathematical model for this type of concentric antagonistic receptive field, which consists of two Gaussian distributions in opposite directions, as expressed below:


(1) $$S\left(x\right)=k_{c}\cdot \exp\left(\frac{-x^{2}}{2r_{c}^{2}}\right)-k_{s}\cdot \exp\left(\frac{-x^{2}}{2r_{s}^{2}}\right)$$

The sensitivity of the excitatory center and the inhibitory surround is, respectively, controlled by $k_{c}\;$ and $k_{s}$ , while the range of influence of the excitatory center and the inhibitory surround is, respectively, controlled by $r_{c}$ and $r_{s}$ , with $r_{c}<r_{s}$ . As it is composed of two mutually antagonistic Gaussian distributions, it is also known as the difference-of-Gaussians model. This spatially antagonistic receptive field is the basis for neural ganglion cells to exhibit lateral inhibition physiologically.

**Figure 1:**
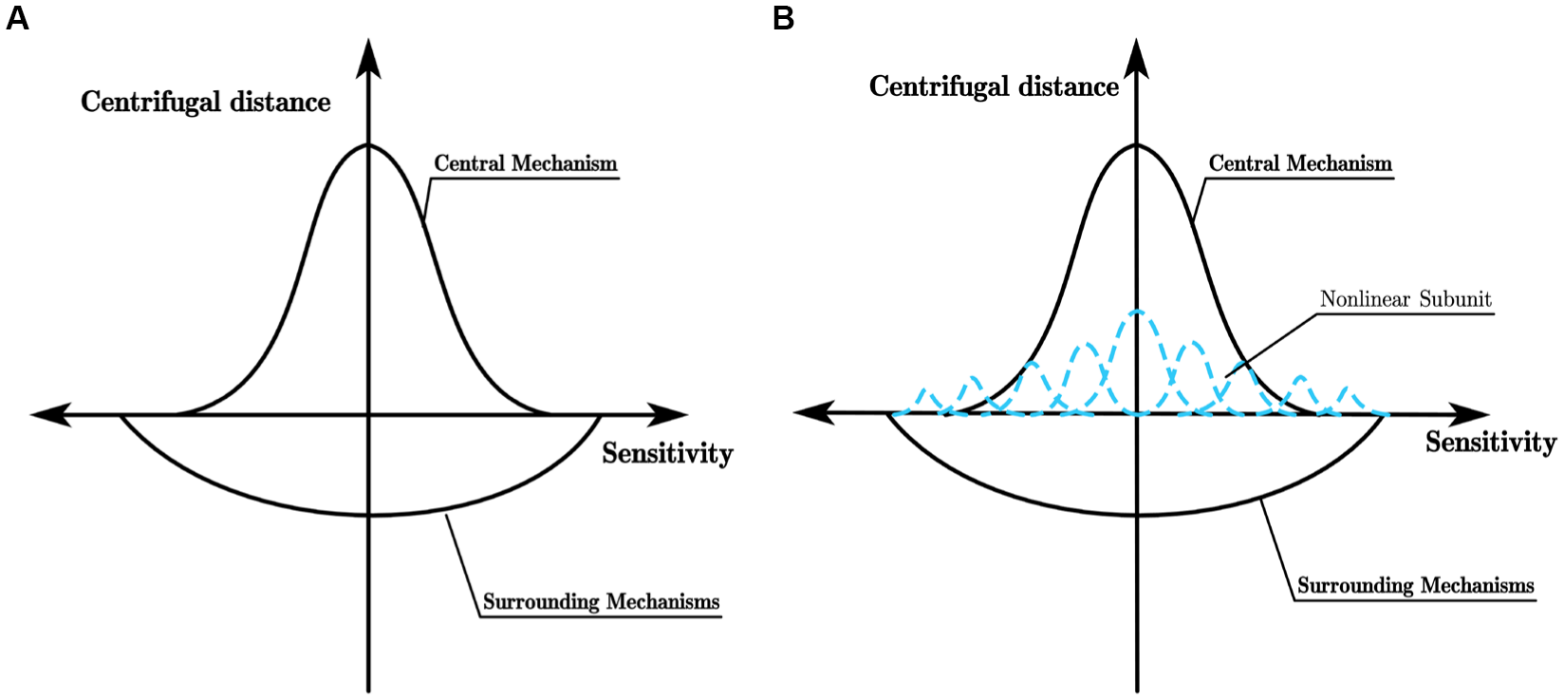
**(A)** Receptive field model of X cell; **(B)** Receptive field model of Y cell.

Enroth-Cugell and Robson (1966) found that while the Gaussian difference model can effectively describe the receptive field of X ganglion cells (referred to as X-cells), it fails to match the experimental data of Y ganglion cells (referred to as Y cells). X cells and Y cells exhibit distinct response patterns to sinusoidal gratings falling within their receptive fields. When the sinusoidal grating causes the intensity of light within the receptive field of the ganglion cell to be equal to the mean light intensity, X cells show almost no response, whereas Y cells produce a strong response to the appearance and disappearance of the sinusoidal grating in their receptive field. This difference in response to stimuli stems from the distinct receptive field characteristics of X cells and Y cells. The excitatory and inhibitory effects of the receptive field of X cells can be linearly superimposed, and their receptive field model can be represented by the Gaussian difference model shown in Figure 1A. In contrast, for Y cells, Hochstein and Shapley’s research (Hochstein and Shapley, 1976a,b) suggests that in addition to having a concentric antagonistic mechanism, their receptive field also contains a non-linear subunit with rectifying properties that is sensitive to the second harmonic component within the receptive field. Such a receptive field model of Y cells is shown in Figure 1B. Similar to retinal ganglion cells, LGN cells also have concentric antagonistic receptive fields and can be divided into X cells and Y cells, playing a crucial role in the transmission and pre-processing of visual information.

**Figure 2:**
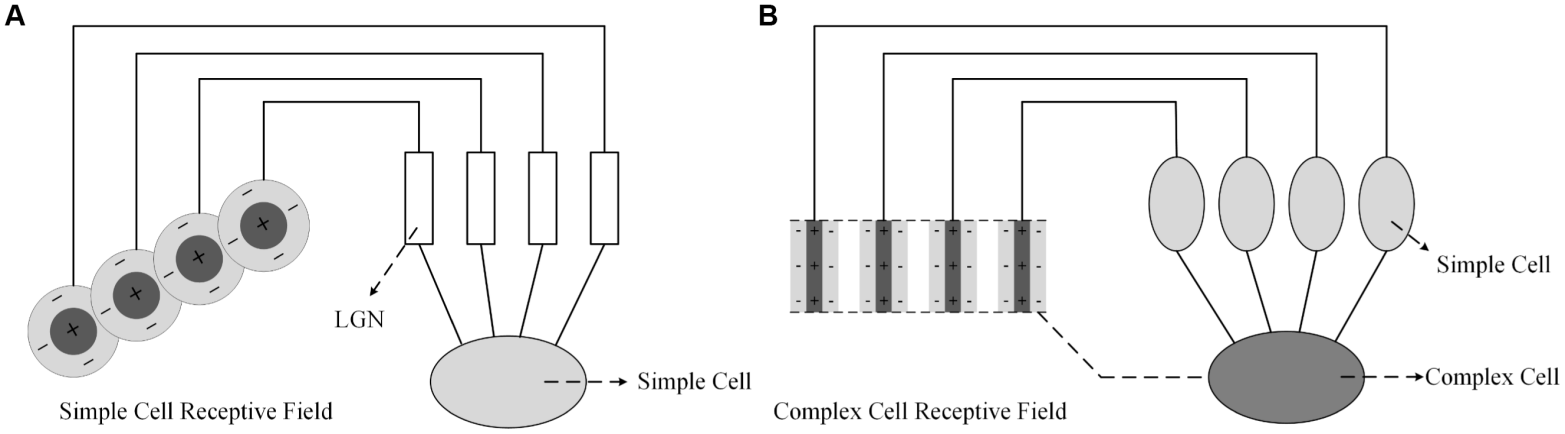
Formation mechanisms of visual cortex cell’s receptive field proposed by Hubel and Wiesel; **(A)** Simple cell; **(B)** Complex Cell.

Hubel and Wiesel (1962, 1965) proposed that the receptive fields of primary visual cortex neurons are formed by the convergence of receptive fields of lower-level neurons. As shown in Figure 2A, the receptive field of a simple cell is composed of multiple LGN cells with concentric receptive fields, which are arranged in a line on the retina, resulting in a narrow receptive field for the simple cell with the line’s orientation being its preferred orientation. As shown in Figure 2B, the receptive field of a complex cell is formed by the convergence of simple cells with bar-shaped receptive fields, which are arranged in a line in space. Therefore, the light and dark edges that satisfy the preferred orientation of the simple cells can cause a response in the complex cell regardless of their location. It is believed (Shou, 1997) that complex cells mainly focus on the abstract concept of orientation in visual information. There is currently little research and conclusion on hypercomplex cells, but it is certain that they have stricter requirements for optimal stimuli and only respond to specific orientations such as breakpoints or corners.

### 3.2. Network architecture

**Figure 3:**
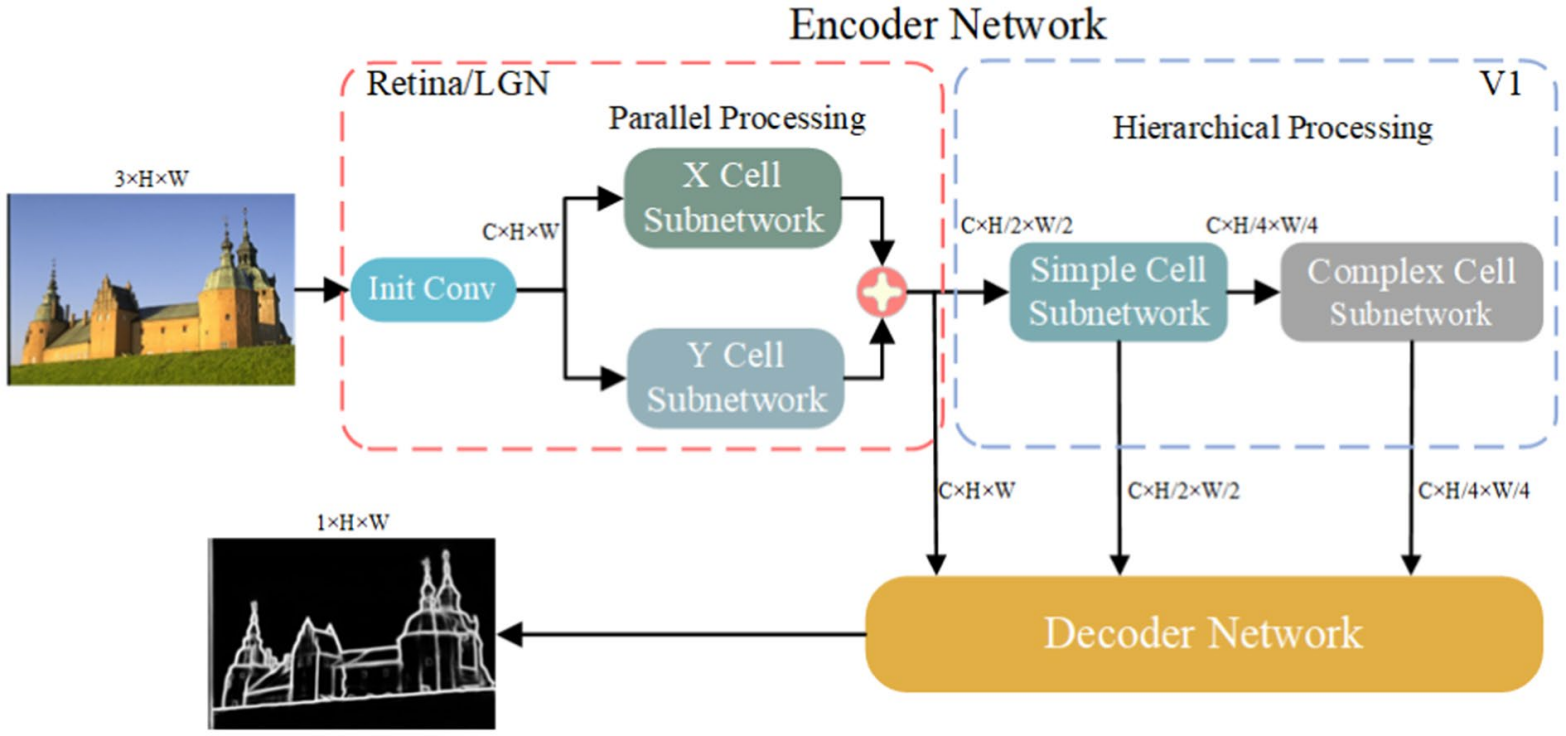
Parallel processing and hierarchical processing network framework.

In biological vision, the formation of edge information occurs in the retina-LGN-V1 pathway. During this process, the parallel processing in the retina and LGN, as well as the hierarchical processing in V1, play important roles in extracting edge information. In the parallel processing, X-type and Y-type cells constitute the X and Y parallel pathways. The hierarchical processing then processes the information based on the hierarchical forms of simple and complex cells. Inspired by this visual information transmission mechanism, we designed a network model with an encoding-decoding structure, as shown in Figure 3.

In the encoding network, there are subnetworks for X cells, Y cells, simple cells, and complex cells, respectively. The design of the X and Y cell subnetworks is inspired by the receptive field properties of corresponding cells in the retina and LGN. Similarly, the design of the simple and complex cell subnetworks is inspired by the receptive field properties of corresponding cells in V1. In the decoding network, the target edges are extracted based on the features extracted through parallel and hierarchical processing.

The Init Conv is a 1×1 convolutional layer used to adjust the input features from 3 channels to C channels. In the parallel processing, the two subnetworks are responsible for extracting contrast information, while in the hierarchical processing, the two subnetworks respond to specific directional features. Furthermore, we increase the receptive field of cells by applying max pooling before the information enters the modules for simple cells and complex cells. Finally, the contour information is formed through the decoding process. The design of the X and Y cell subnetworks is detailed in Section “X-cell and Y-cell subnetwork”, the design of the simple and complex cell subnetworks is described in section “Simple and complex cells subnetwork”, and the structure of the decoding network is explained in section “Decoding network”.

#### 3.2.1. X-cell and Y-cell subnetwork

**Figure 4:**
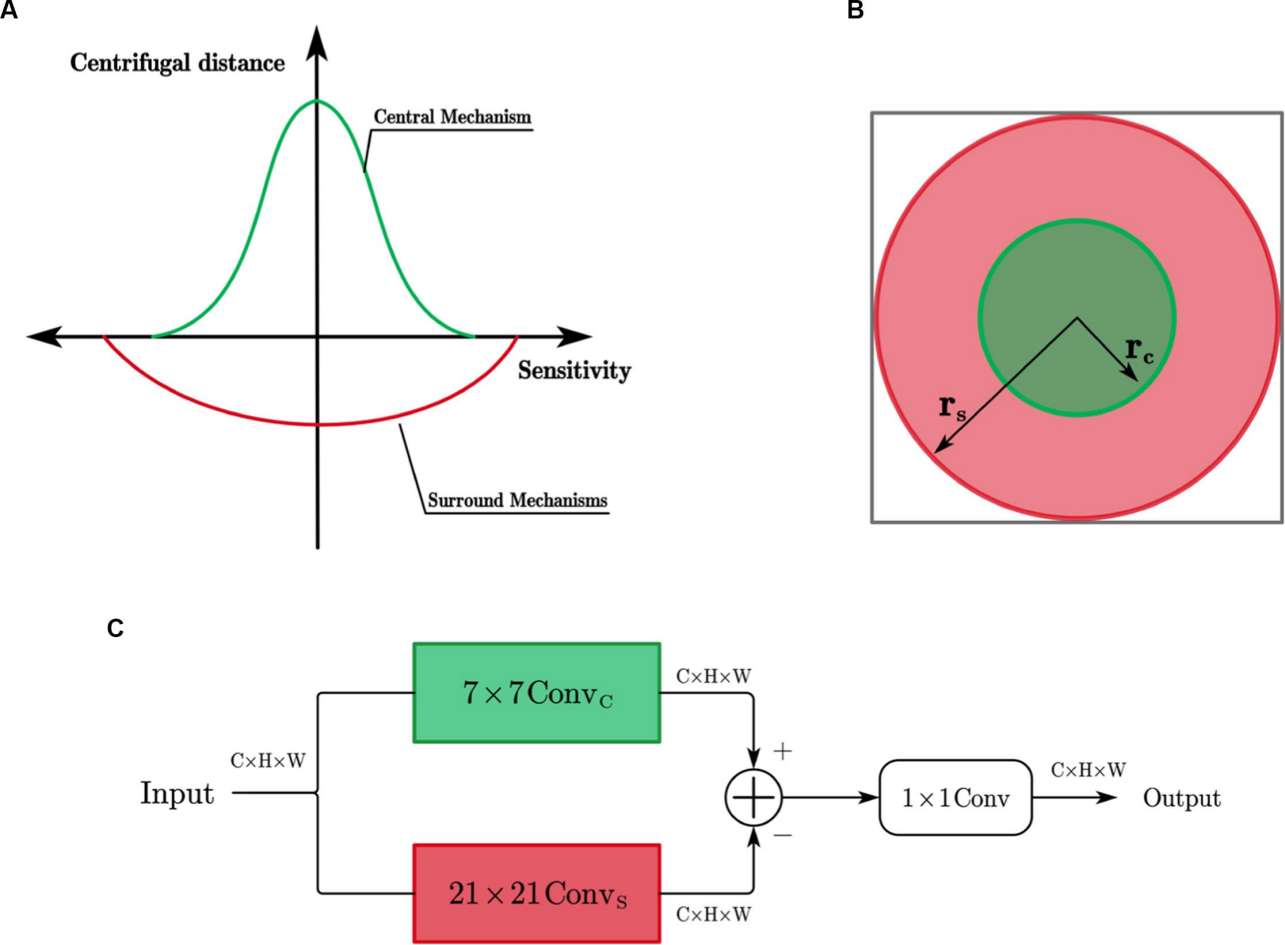
**(A)** Simulation of X cell receptive field; **(B)** Extent of the central and surrounding mechanisms; **(C)** X cell subnetwork.

As shown in Figure 4A, X cells have concentric antagonistic receptive fields. Traditional biomimetic edge detection methods directly use the Gaussian difference model proposed by Rodieck (1965) to simulate the receptive field of X cells. They use fixed Gaussian function templates to simulate the contributions of the center and surround mechanisms to cell responses. In contrast to these methods, this study uses learnable convolutional kernels to simulate the receptive field of X cells.

The ranges of the center and surround mechanisms’ effects on cell responses are illustrated in Figure 4B and contribute with opposite signs. The center response mechanism is represented by a 7 × 7 depth-wise separable convolutional layer ( ${\mathrm{Conv}}_{C}$ ), while the surround response mechanism is composed of a 21 × 21 depth-wise separable convolutional layer ( ${\mathrm{Conv}}_{S}$ ). The effective receptive field is constructed as a circular ring, and the antagonistic mechanism is formed by subtracting the surround response from the center response.

The X-cell subnetwork structure, inspired by the center-surround antagonism mechanism in X cells, is depicted in Figure 4C of this paper. When given an input feature F with n channels, the convolution operation is performed channel-wise using the center-surround mechanism of X cells. The mathematical expression for the calculation process is as follows:


(2) $$R_{c}^{i}=\mathrm{ReLU}\left(\mathrm{Norm}\left(F^{i}*K_{c}^{i}\right)\right)$$

(3) $$R_{s}^{i}=\mathrm{ReLU}\left(\mathrm{Norm}\left(F^{i}*K_{s}^{i}\right)\right)$$

Where i∈{1,2,⋯,n} represents the channel index, $R_{c}^{i}$ denotes the feature of the *i*-th channel after the center mechanism, and $R_{s}^{i}$ represents the feature of the *i*-th channel after the surround mechanism. $K_{c}^{i}$ denotes the convolution kernel ( ${\mathrm{Conv}}_{C}$ ) that simulates the center mechanism, while $K_{s}^{i}$ represents the convolution kernel ( ${\mathrm{Conv}}_{S}$ ) that simulates the surround mechanism. These kernels are circular rings with an inner radius of $r_{c}$ and an outer radius of $r_{s}$ . ReLU(⋅) denotes the activation function, and Norm(⋅) represents the normalization operation.

The center and surround responses have opposite contributions, meaning they exhibit antagonistic. The antagonistic response of the X-cell subnetwork is obtained by calculating the difference between the features obtained after the responses of the center and surround mechanisms. The calculation process is as follows:


(4) $$E_{X}^{i}=R_{c}^{i}-\omega \cdot R_{s}^{i}$$

Where $E_{X}^{i}$ represents the antagonistic response of the *i*-th channel, ω is a learnable weight parameter that controls the strength of the surround mechanism’s inhibition on the center mechanism. After obtaining the antagonistic response of each channel of the X cells, this paper employs a standard 1×1 convolution to integrate the responses across all channels, resulting in the final response of the X cells.

**Figure 5:**
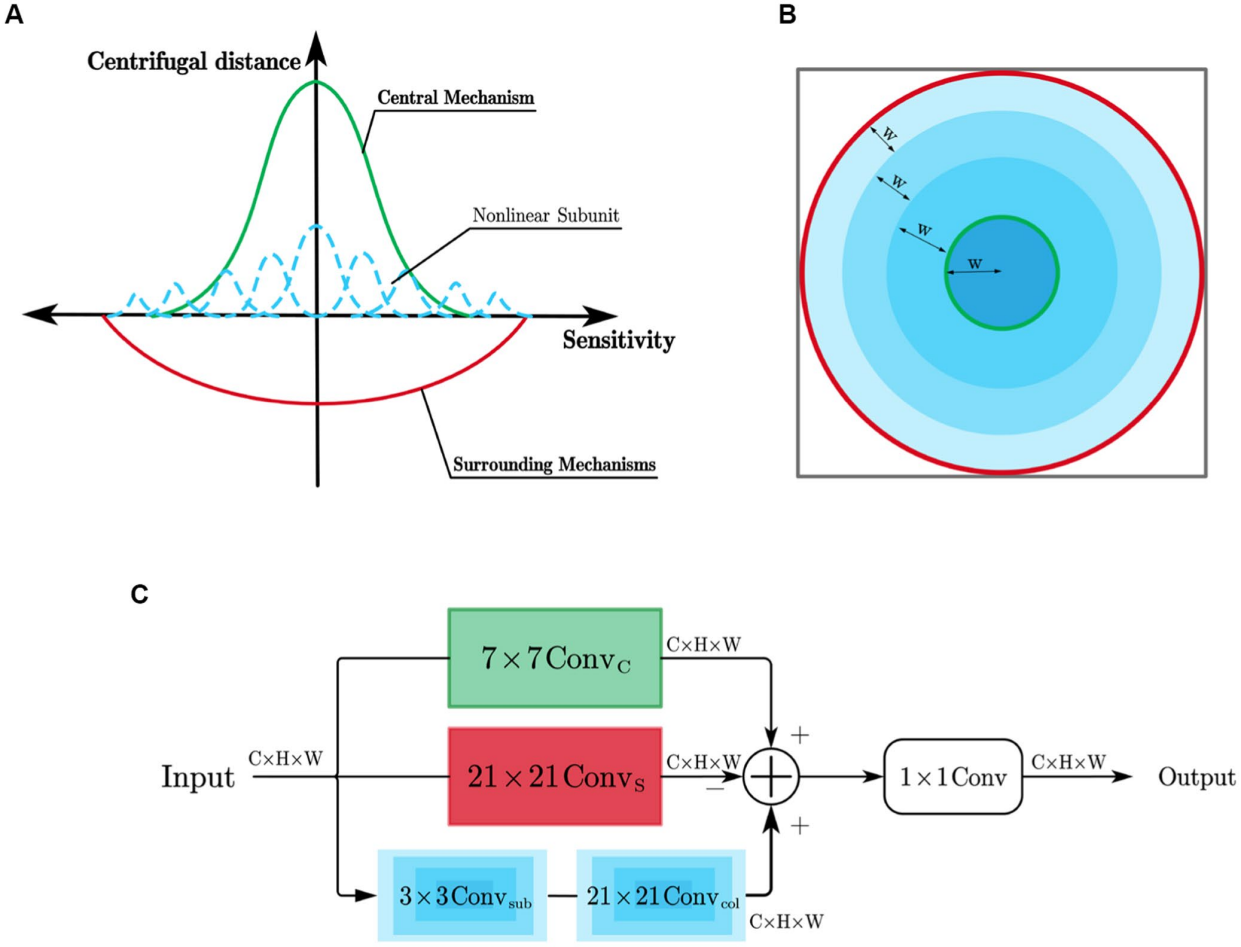
**(A)** Simulation of Y cell receptive field; **(B)** Manner of partitioning the area of responsibility of the non-linear subunits; **(C)** Y cell subnetwork.

Y cells’ receptive field, in addition to having the same center and surround mechanisms as X cells, also possess non-linear subunits that exhibit rectification properties, as shown in Figure 5A. The receptive field of Y cell models is simulated using learnable convolutional kernels. The simulation of the center-surround mechanism in the Y cell model is the same as that in the X cell model, but the Y cell model incorporates unique non-linear subunit models.

The contributions of the non-linear subunits to the cell’s response have the same sign as the center mechanism and have a distribution range that is roughly similar to the surround mechanism. This paper considers that the differences between the non-linear subunits depend on their distances from the receptive field center. Therefore, the influence range of the non-linear subunits is divided into multiple concentric circular regions using a certain spacing denoted as W, forming a depth-wise separable convolution ( ${\mathrm{Conv}}_{\mathrm{col}}$ ) with multiple differently sized circular rings. The construction method of these circular rings is consistent with the surround convolution method of X cells. By adjusting the radius, the size of each circular ring can be controlled, and each region is responsible for a specific type of non-linear subunit. Each non-linear subunit is composed of a 3×3 depth-wise separable convolution ( ${\mathrm{Conv}}_{\mathrm{sub}}$ ). In the example shown in Figure 5B, the influence range of the non-linear subunits is divided into four regions, each corresponding to a different type of non-linear subunit.

The structure of the Y-cell subnetwork is shown in Figure 5C. For an input feature F containing *n* channels, the Y-cell subnetwork performs a channel-by-channel convolution of it and obtains the Y cell response by integrating the pericentral mechanism response with the overall response of the nonlinear subunits, since the pericentral antagonistic response mechanism remains the same as the X cell subnetwork computational process. The Y-cell subnetwork computational process is as follows:


(5) $$E_{Y}^{i}=E_{X}^{i}+R_{NLU}^{i}$$

The computation process for obtaining $R_{NLU}^{i}$ , which represents the feature of the *i*-th channel after the response of the non-linear subunit, is as follows:


(6) $$R_{NLU}^{i}=\frac{1}{k}\overset{k}{\underset{j=1}{\sum }}ReLU\left(Norm\left(F^{i}*K_{sub}^{i,j}\right)\right)*K_{col}^{i,j}$$

Where k represents the number of non-linear subunits, $K_{sub}^{i,j}$ denotes the learnable convolutional kernel for the *j*-th non-linear subunit, and $K_{col}^{i,j}$ is responsible for collecting the response of the *j*-th non-linear subunit in its corresponding region to the central position. It has a ring-shaped structure with a width of *w* and operates in a serial manner with the convolution operation of the non-linear subunit. After obtaining the Y-cell responses of each channel, the conventional 1 × 1 convolution is used in this paper to integrate each channel to obtain the final Y-cell responses.

#### 3.2.2. Simple and complex cells subnetwork

**Figure 6:**
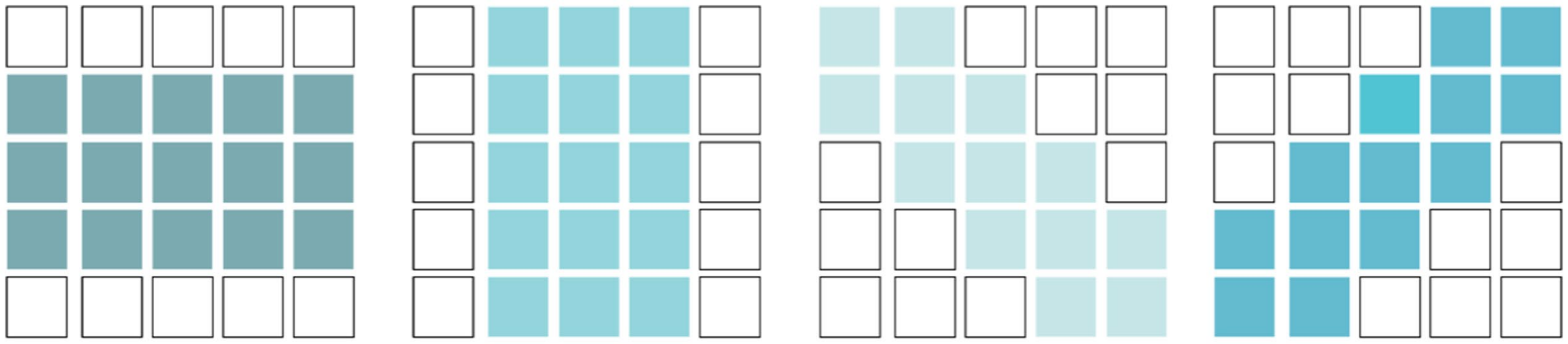
Convolutional Kernels simulating orientation-selective receptive fields.

Simple cells have elongated receptive fields and exhibit orientation selectivity. To simulate this, we used learnable orientation-selective convolutional kernels with four different directions, as shown in Figure 6. For input feature F with n channels, we processed it channel-wise. The response of the *i*-th simple cell, denoted as $E_{s}^{i}$ , can be expressed as follows:


(7) $$E_{s}^{i}=\frac{1}{4}\overset{4}{\underset{j=1}{\sum }}\mathrm{ReLu}\left(Norm\left(MaxPool\left(F^{i}\right)*K_{ori}^{i,j}\right)\right)$$

Here, MaxPool(⋅) represents a 2×2 pooling operation, which is used to enlarge the receptive field of the simple cells. $K_{ori}^{i,j}$ is the learnable convolutional kernel of the *i*-th simple cell in the *j*-th direction. After obtaining the response of each channel’s simple cells, we integrated them using a 1 × 1 convolution to obtain the final response of the simple cells.

**Figure. 7:**
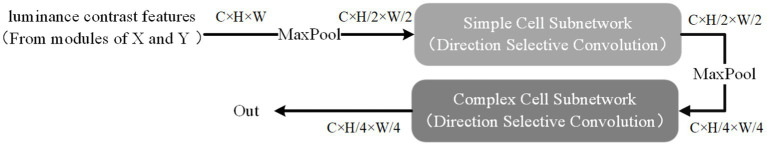
Hierarchical processing.

There is no precise description of the receptive field of complex cells. However, it is certain that they have elongated receptive fields, similar to those of simple cells. As mentioned earlier, the receptive field of a complex cell is formed by the convergence of receptive fields of several simple cells, and the difference between simple and complex cells may be dynamically reflected in the visual cortex cells. Therefore, in this article, the receptive field of complex cells is not directly modeled. Instead, we use direction-selective convolution kernels, the same as those used for simple cells, to simulate the receptive field of complex cells. The convergence of the receptive field of complex cells to that of simple cells is represented through a hierarchical processing approach. The hierarchical network consisting of simple cell models and complex cell models is shown in Figure 7.

#### 3.2.3. Decoding network

**Figure 8:**
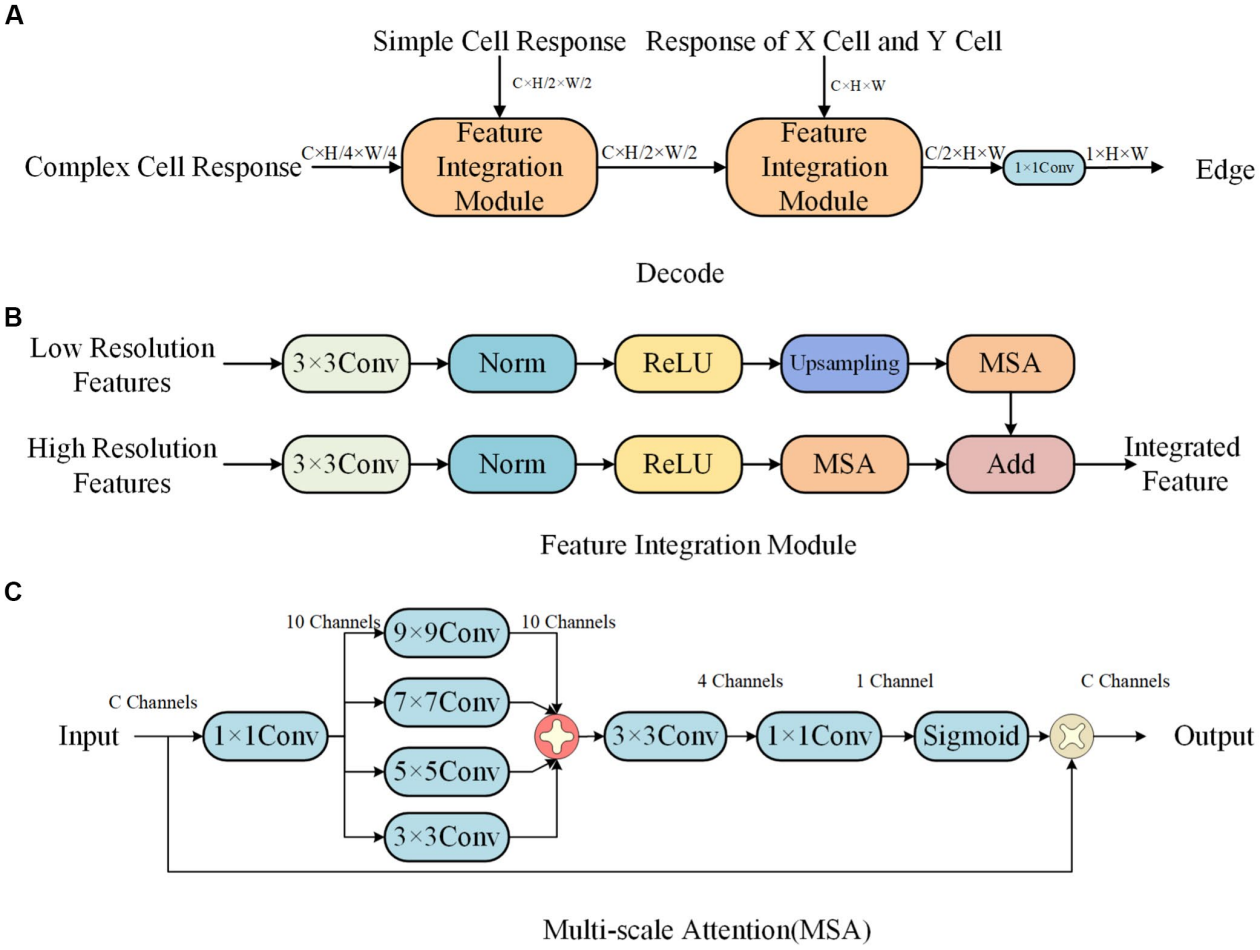
Decode network structure. **(A)** Decode. **(B)** Feature Integration module. **(C)** Multi-scale attention (MSA).

The decoding network has a structure as shown in Figure 8A, and the feature integration module is shown in Figure 8B. It uses a progressive upsampling approach to integrate features of different sizes, effectively ensuring the accuracy of edge information. In order to better focus on edge information and reduce the influence of surrounding texture information, we propose a multi-scale attention mechanism, as shown in Figure 8C.

## 4. Experiment

In this section, we conducted a performance evaluation of the proposed model based on the BSDS500 and MBDD datasets. We analyzed the model from three aspects: accuracy, computational complexity, and parameter quantity, and provided an assessment of its strengths and weaknesses.

### 4.1. Experimental details

In the proposed encoding network, the number of channels in the feature maps was set to 32, and the sizes of all the center and surround mechanism convolutional kernels were set to 7 and 21, respectively. For the Y-cell model, the size of the non-linear subunit convolutional kernel was set to 3×3, and the number of kernels responsible for the annular region was set to 5, with a width of 4.

During the model training process, a relatively large learning rate was used to train the model, and gradient clipping was employed to overcome the problem of exploding gradients. Gradient clipping refers to the practice of limiting the values of large gradients in a deep neural network during the training process, to prevent the learned parameters from being updated in a direction that produces even larger gradients. Specifically, in this paper, the L2 norm of the gradients was used to limit the gradients of the parameters. After gradient clipping, the gradients for a set of parameters G were restricted to a certain maximum value.


(8) $$G^{\prime }=\left\{\begin{array}{c} G,\;\;\;∥G{∥}_{2}<n\\ \frac{G}{∥G{∥}_{2},}∥G{∥}_{2}\geq n\; \end{array}\right\}$$

The symbol $∥G{∥}_{2}$ represents the L2 norm of the gradient *G*, and *n* is the threshold value set for the gradient. It is evident that when the L2 norm of the gradient exceeds the threshold value, the actual gradient used for updates will be scaled down to a smaller value, otherwise no operation is required on the gradient.

We implemented, trained, and tested our proposed network using the PyTorch (Imambi et al., 2021) deep learning framework. All convolutional kernels in the network were randomly initialized with a Gaussian distribution with a mean of 0 and a variance of 0.02. We used the AdamW (Loshchilov and Hutter, 2017) optimizer for training the model with an initial learning rate of 1 × 10–3 and processed only one image per data iteration. The loss function is consistent with the literature (Su et al., 2021).

We evaluate our model on the BSDS500 and MBDD datasets. On the BSDS500 dataset, the model undergoes 10 iterations on all training data, and the learning rate is reduced to 0.1 times the previous iteration’s after completion of the 9th iteration. On the MBDD dataset, the model undergoes 7 iterations on all training data, and the learning rate is reduced to 0.1 times the previous iteration’s after completion of the 4th and 6th iterations. We use the standard non-maximum suppression algorithm to refine the output edges when evaluating the model’s performance.

All our experiments were conducted on a server with the following specifications: a Windows 10 operating system, an lntel(R) Xeon(R) Silver 4210R CPU, an NVIDIA GeForce 3,090 GPU, and 128GB of Random-access memory (RAM).

### 4.2. Evaluation standards

In the evaluation of edge detection algorithms, researchers often use the F-score as a performance metric in classification tasks. Martin et al. (2004) employed the F-score to evaluate the performance of various algorithms in edge detection. The F-score is calculated using the following formula:


(9) $$F=\frac{P\cdot R}{\left(1-\alpha \right)\cdot P+\alpha \cdot R}$$

In the equation, P represents the precision rate, R represents the recall rate, and α is the weight that balances the contribution of Precision and Recall. Typically, α is set to 0.5. P and R are calculated as follows:


(10) $$P=\frac{TP}{TP+FP}$$

(11) $$R=\frac{TP}{TP+FN}$$

Here, TP refers to the number of pixels predicted as edges that are actually edges, FP refers to the number of pixels predicted as edges that are not edges, and FN refers to the number of pixels predicted as non-edges that are actually edges.

**Figure 9:**
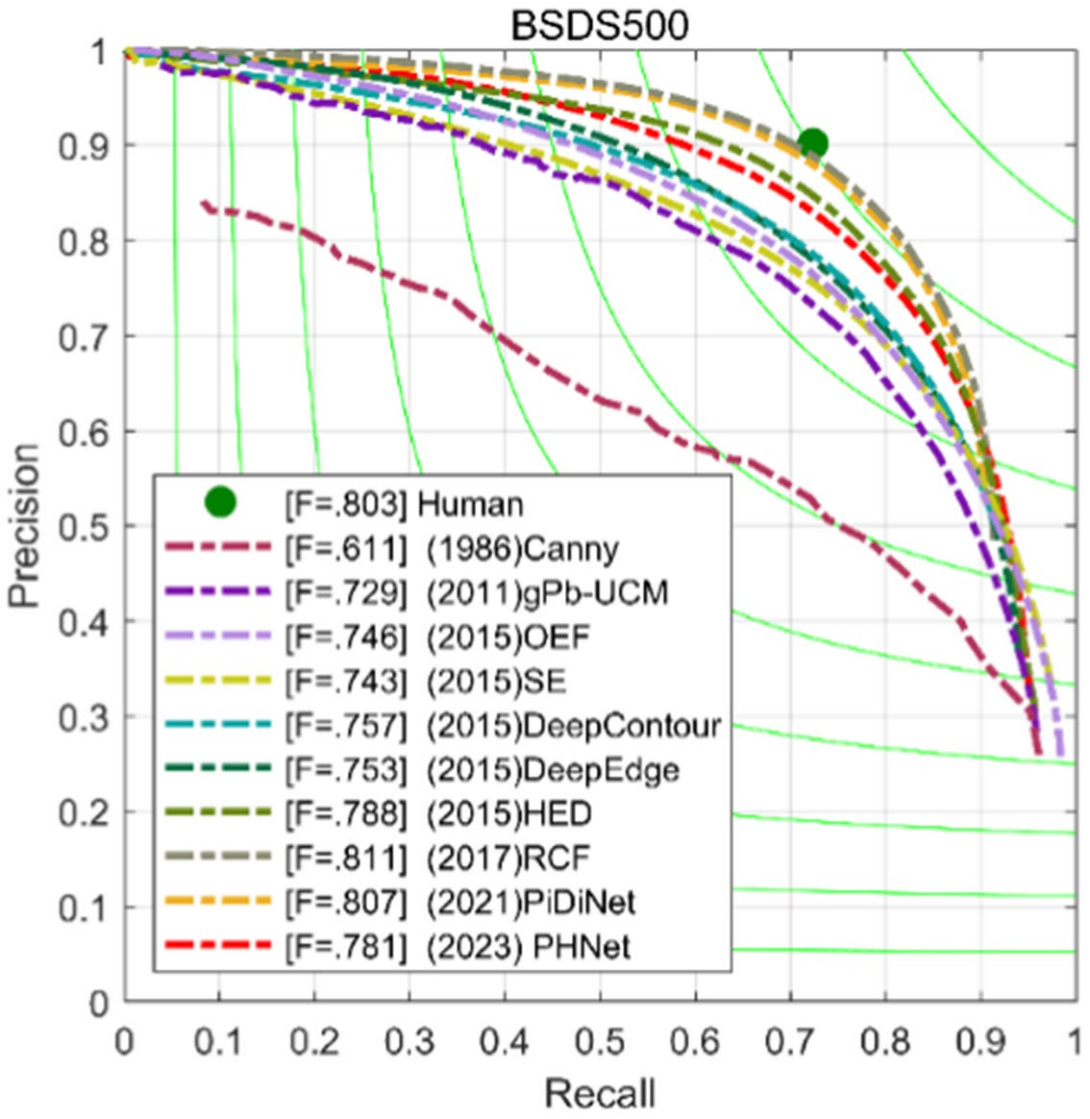
Precision-recall curves of our models and some competitors on BSDS500 dataset.

Furthermore, the Precision-Recall (PR) curve is a graphical representation with Precision on the *Y*-axis and Recall on the *X*-axis, as shown in Figure 9. Higher Precision and Recall values indicate better algorithm performance. In other words, a PR curve located closer to the upper right corner of the graph indicates better model performance, and the area under the curve should be maximized.

The choice of threshold during the binarization process significantly affects the evaluation results of edge detection algorithms. This means that selecting a reasonable threshold is crucial for evaluating algorithm performance. To ensure fair and reasonable performance evaluation, Arbelaez et al. (2010) proposed three performance measures: Optimal Dataset Scale (ODS) and Optimal Image Scale (OIS). ODS involves setting the same threshold for all images in the dataset to maximize the *F*-score across the entire dataset, reflecting the overall algorithm performance. OIS involves setting different thresholds for each image to maximize the F-score on each image individually and then taking the average, reflecting the algorithm’s best performance across different images.

In addition to evaluating the model using ODS F-measure and OIS F-measure, this paper also analyzes the model using Floating Point Operations (FLOPs) and parameter count (Params). FLOPs measure the computational complexity of the model by counting the number of floating-point operations performed when processing data. Each multiplication or addition of floating-point numbers in the model is counted as one operation. In this paper, the calculation of FLOPs for convolutional layers without biases is as follows:


(13) $$FLOPs=2\times C_{i}\times K^{2}\times H\times W\times C_{o}$$

where $C_{i}$ is the number of input channels, K is kernel size, H and W are the height and width of the input features, and $C_{o}$ is the number of output channels. To ensure fair comparison and convenience, in this paper, the FLOPs are uniformly calculated using H=320,W=320 as the dimensions. Params represent the number of trainable parameters in the model. For example, in the Y cell subnetwork, the parameter count of a 32-channel 3×3 non-linear subunit convolutional kernel is 32 * 3 * 3 = 228.

### 4.3. Ablation experiments

In order to investigate the impact of different modules proposed in this study on the model’s performance, we conducted ablation experiments on the BSDS500 dataset. The results are presented in Table 1. The models *X*, *Y*, and *S* represent the X-cell model, Y-cell model, and direction-selective convolution model (simulating simple and complex cells), respectively. The “✔” indicates that the corresponding module is used in the model, while the “✘” indicates that the traditional convolution is used instead. It can be seen that when the network uses only the X-cell model, Y-cell model, or direction-selective convolution model S separately, there is an improvement in ODS compared to the traditional convolution method. However, when all three modules proposed in this study are applied to the network, ODS further improves. This indicates that the three modules proposed in this study contribute to improving the edge detection performance of the parallel hierarchical network.

**Table 1:** Results of ablation experiments with different modules on BSDS500 dataset.

X model	Y model	S model	ODS	OIS
✘	✘	✘	0.768	0.786
✔	✘	✘	0.772	0.792
✘	✔	✘	0.774	0.793
✘	✘	✔	0.778	0.796
✔	✔	✔	0.781	0.797

To investigate the impact of the proposed multi-scale attention module on the model, we conducted an ablation experiment by removing the attention module while keeping all other experimental details the same. The results are recorded in Table 2. It is evident from the results that the multi-scale attention significantly improves the edge detection performance, indicating the effectiveness of this module in the proposed network.

**Table 2:** Results of ablation experiments with Multi-scale attention (MSA) on BSDS500 dataset.

MSA	ODS	OIS
✘	0.775	0.793
✔	0.781	0.797

### 4.4. BSDS500 dataset experiment

The BSDS500 dataset is widely used for evaluating the performance of edge detection models, which includes 200 training images, 100 validation images, and 200 testing images. Due to the limited amount of training data, this paper uses data augmentation on the 200 training images and 100 validation images, and also expands the training data with the PASCAL VOC dataset.

This paper compares PHNet with several methods, including transfer learning-based methods(TL-based methods) such as HED (Xie and Tu, 2017), RCF (Liu et al., 2017), BDCN (He et al., 2019); lightweight deep learning methods such as TIN (Wibisono and Hang, 2020b), FINED (Wibisono and Hang, 2020a), and PiDiNet (Su et al., 2021); bio-inspired methods such as SED (Akbarinia and Parraga, 2018), SCO (Yang et al., 2015; Tang et al., 2019); and non-deep learning methods such as gPb (Arbelaez et al., 2010), OEF (Hallman and Fowlkes, 2015), SE (Dollár and Zitnick, 2014). The results are recorded in the Table 3 and Figure 9.

**Table 3:** Evaluation results on BSDS500 dataset.

Type	Method	ODS	OIS	Params	FLOPs
TL-base method	HED (Xie and Tu, 2017)	0.788	0.808	14.72 M	31.4G
	RCF (Liu et al., 2017)	0.806	0.823	15.51 M	36.6G
	BDCN (He et al., 2019)	0.820	0.838	16.30 M	56.0G
	BDCN2 (He et al., 2019)	0.766	–	0.28 M	22.6G
	BDCN3 (He et al., 2019)	0.796	–	2.26 M	34.0G
Lightweight method	TIN (Wibisono and Hang, 2020b)	0.772	0.795	0.24 M	12.9G
	FINED (Wibisono and Hang, 2020a)	0.790	0.808	1.43 M	29.3G
	PiDiNet (Su et al., 2021)	0.807	0.823	0.72 M	8.3G
Biology-inspired method	SED (Akbarinia and Parraga, 2018)	0.710	0.740	–	–
	SCO (Yang et al., 2015)	0.670	0.710	–	–
	Tang (Tang et al., 2019)	0.762	0.778	–	–
Non-deep Learning method	gPb (Arbelaez et al., 2010)	0.729	0.755	–	–
	OEF (Hallman and Fowlkes, 2015)	0.746	0.770	–	–
	SE (Dollár and Zitnick, 2014)	0.743	0.764	–	–
Biology-inspired Lightweight method	PHNet(Our)	0.781	0.797	0.20 M	16.0G

Table 3 shows the performance of each model in terms of ODS, OIS, parameter count Params, and computational complexity FLOPs at a single scale. PHNet has some competitiveness in ODS and OIS compared to other models, and it has the least number of parameters compared to other methods, indicating that PHNet can effectively use convolutional kernel parameters and save computational resources.

Compared with HED, RCF, and BDCN, which are based on transfer learning, these models use VGG16 as the encoding network for edge detection. The performance improvement from HED to RCF and then to BDCN comes at the cost of increasing Params and FLOPs. These methods improve edge detection performance by designing increasingly complex decoding networks. Compared with RCF, PHNet has 3.1 and 3.2% lower ODS and OIS performance indicators, respectively, but only requires 1.3% of its Params and 43.7% of its FLOPs. Compared with BDCN, PHNet has 4.8 and 4.9% lower ODS and OIS performance indicators, respectively, but its Params and FLOPs are only 1.2 and 28%, respectively.

BDCN tests the model’s performance by gradually reducing the number of network modules when using fewer parameters. The specific method is to gradually remove the deep modules of the VGG16 backbone network. BDCN3 is a network variant of BDCN that only retains the first three stages of the VGG16 backbone network. In comparison, PHNet is only 1.9% lower in ODS, and its Params and FLOPs are only 6.2 and 35%, respectively. Comparing PHNet with BDCN2, it is found that as the number of network modules in the BDCN method decreases, PHNet’s ODS has already begun to surpass BDCN method, with a difference of 2.8%, while PHNet still has fewer Params and smaller FLOPs. In general, the comparison with HED, RCF, and BDCN methods shows that edge detection methods based on transfer learning have a large amount of parameter redundancy, and their utilization efficiency of parameters is very low.

**Figure 10:**
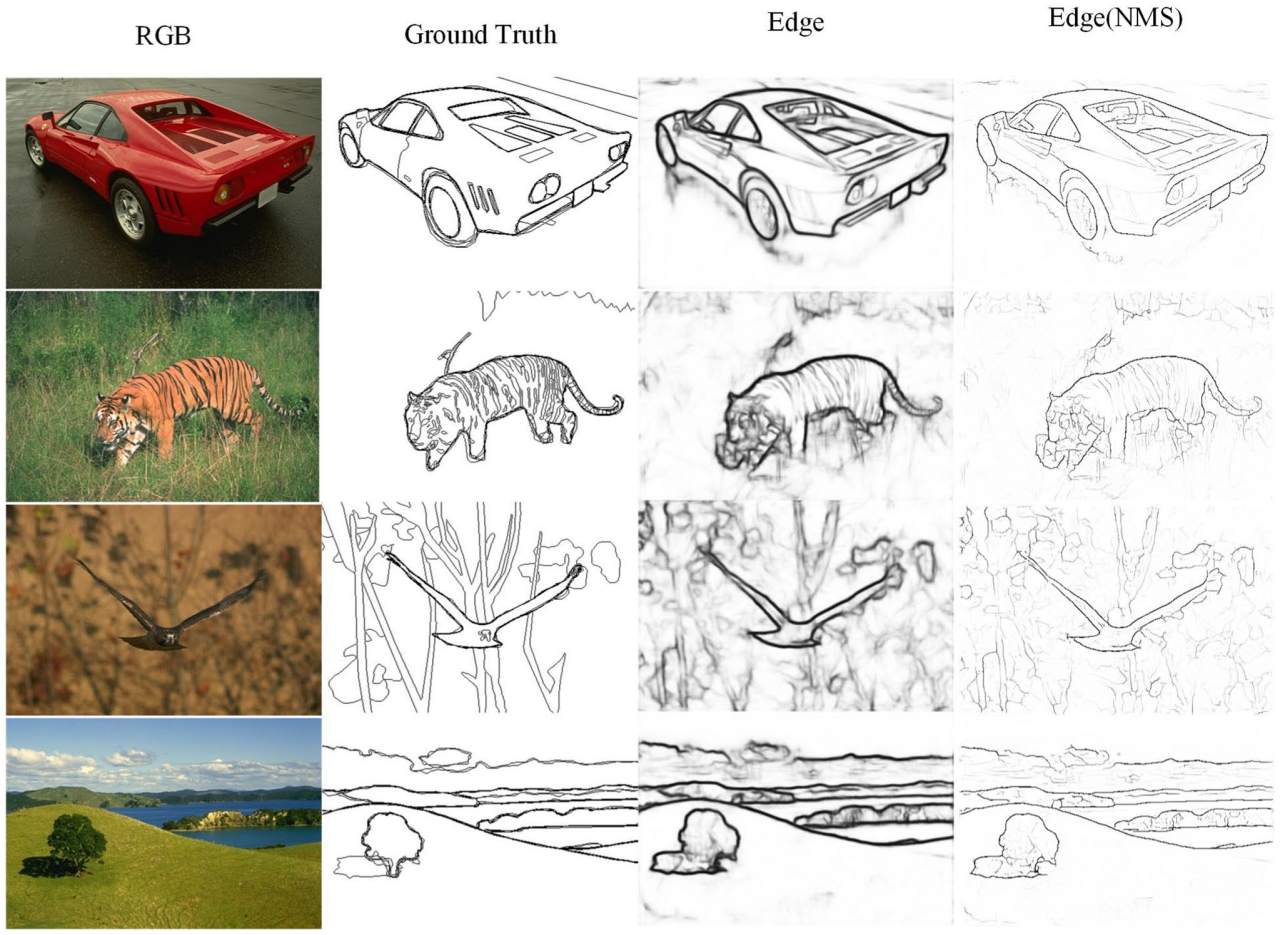
Visualization of contour detection results on BSDS500 dataset from PHNet.

The TIN, FINED, and PiDiNet models are lightweight edge detection neural network models designed to reduce model parameter count and computational complexity. It can be observed that the Params of these networks differ at least an order of magnitude compared to traditional networks. Compared to TIN, PHNet excels in both edge accuracy performance metrics and model parameter count and computational complexity, surpassing TIN by 1.2 and 0.2% in ODS and OIS, respectively, with Params accounting for 83.3% of the difference. Compared to FINED, PHNet achieves similar ODS performance with lower parameter count. As for PiDiNet, the trade-off between model performance and complexity makes it difficult to determine which model is better. It should be noted that in the implementation of the network models, rectangular and annular convolution kernels were used, and any excess parts were included in the calculation of Params and FLOPs. Therefore, PHNet’s actual Params and FLOPs are expected to be even lower. When implemented using lower-level code, the advantages of PHNet in terms of parameter count and computational complexity will be even greater. Figure 10 shows some edge detection results of PHNet model in the BSDS500 dataset, where NMS represents the non-maximum suppression post-processing of the edge output. It can be observed that PHNet’s ability to suppress texture components is still insufficient.

In conclusion, this paper presents a model that achieves good performance with a low parameter count, thanks to the powerful fitting capabilities of convolutional neural networks and the effectiveness of biological vision mechanisms.

### 4.5. MBDD dataset experiment

The MBDD dataset, also known as the Multi-cue Boundary Detection Dataset, comprises 100 stereo video sequences captured by binocular cameras, with a unified resolution of 1280×720. For each video sequence, low-level edges and object boundaries are annotated for the last frame of the left part of the sequence. Following the same methodology as other works (Su et al., 2021), we randomly divided the 100 images into 80 training images and 20 testing images, and performed data augmentation on the training images using the BSDS500 dataset enhancement method. The data was then independently partitioned three times, and 20 testing images were used for performance evaluation, with the average of the three evaluation results being taken as the final result.

Table 4 compares the performance of our model with Multicue (Mély et al., 2016), HED (Xie and Tu, 2017), RCF (Liu et al., 2017), and PiDiNet (Su et al., 2021) in terms of ODS, OIS, parameter quantity (Params). The variance of the three evaluation results is indicated in brackets for ODS and OIS. In the low-level edge experimental data, PHNet exhibited excellent performance in terms of ODS and OIS. Moreover, when considering Params and FLOPs, PHNet only surpassed HED and RCF in their respective detection capabilities for low-level edges by approximately 1% in terms of parameter quantity. Compared to PiDiNet, PHNet led by 0.9% in terms of ODS with only 28% of its parameter quantity, and by 1.4% in terms of OIS. The variance data also indicated that our model exhibited excellent stability in low-level data. Therefore, these results suggest that PHNet exhibited excellent performance in the low-level data of the MBDD dataset. However, in the object boundary experimental data, PHNet exhibited a significant lag compared to other models, which is consistent with our analysis of PHNet’s texture suppression ability in the BSDS500 experiment.

**Table 4:** Evaluation results on MBDD dataset.

Method	Low-level edge		Object boundary		Params
	ODS	OIS	ODS	OIS	
Human	0.750 (0.024)	–	0.760 (0.017)	–	–
Multicue (Mély et al., 2016)	0.830 (0.002)	–	0.720 (0.014)	–	–
HED (Xie and Tu, 2017)	0.851 (0.014)	0.864 (0.011)	0.814 (0.011)	0.822 (0.008)	14.72 M
RCF (Liu et al., 2017)	0.857 (0.004)	0.862 (0.004)	0.817 (0.004)	0.825 (0.005)	15.51 M
PiDiNet (Su et al., 2021)	0.855 (0.007)	0.860 (0.005)	0.818 (0.003)	0.830 (0.005)	0.72 M
PHNet (Our)	0.863 (0.002)	0.872 (0.002)	0.773 (0.003)	0.791 (0.007)	0.2 M

**Figure 11:**
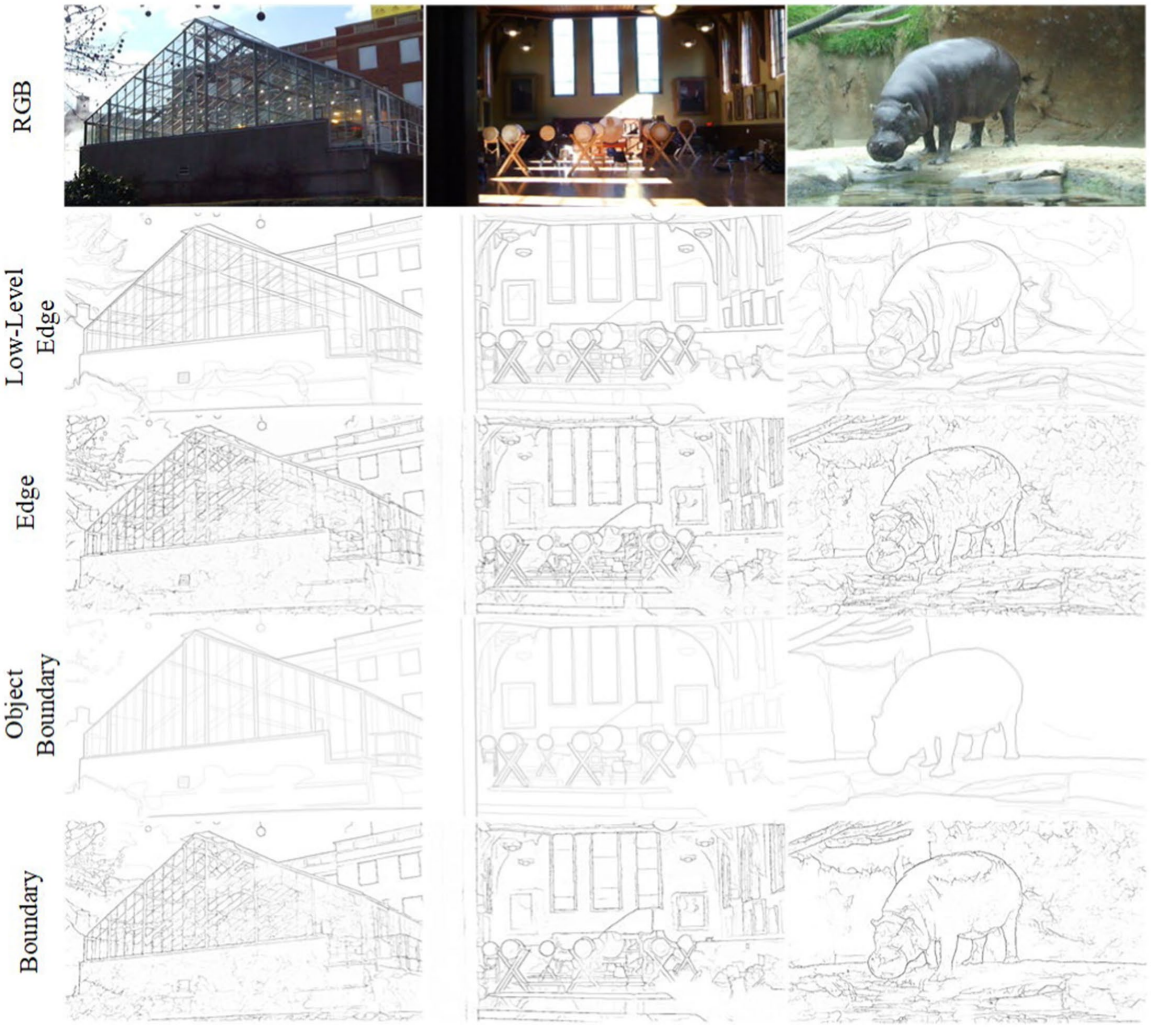
Visualization of testing results on MBDD dataset from PHNet. The first row is the original image, the second row is the Low-level edge training label, the third row is the output of the model trained with the Low-level edge, the fourth row is the Object boundary training label, and the fifth row is the output of the model trained with the Object boundary.

It seems understandable that PHNet model exhibits this phenomenon in Figure 11, because it models the neural activity in the visual cortex at a micro level, simulating the one-way forward processing of visual information. However, visual cortical neurons not only receive feedforward signals, but also receive feedback signals from higher-level visual cortices. These feedback signals rely on extensive inter-projection between different visual cortices, and can regulate the physiological activity of lower-level visual neurons to optimize their information processing.

## 5. Conclusion and prospects

Taking the sacrifice of a small amount of network performance in exchange for lower computational costs as a starting point, this article proposes a lightweight edge detection deep learning model, which utilizes a parallel hierarchical visual information processing mechanism in the simulated visual cortex neurons. The model achieves a more detailed simulation of the shape and response mechanisms of cell receptive fields through large trainable convolution kernels. Circular and annular trainable convolution kernels are used to simulate the center-surround mechanism of X and Y cells receptive fields, and the antagonistic properties of convolution responses are reflected by subtraction. Small convolution kernels and annular trainable convolution kernels are used to simulate the non-linear subunit properties of Y cells, and the contribution of non-linear subunits to the center mechanism is realized by cascading combinations. Narrow trainable convolution kernels with different orientations are used to simulate the direction selectivity of simple cell receptive fields. These convolution models form the lightweight edge detection network PHNet proposed in this paper.

We tested the edge detection performance of the PHNet model on the BSDS500 and MBDD datasets and found that our model can achieve competitive performance with very few parameters compared to some traditional edge detection networks. It also has higher parameter utilization efficiency, which can reserve more computational and storage resources for other visual processing tasks on resource-constrained platforms.

Furthermore, experimental results reveal certain limitations of the PHNet model, including the lack of ability to integrate visual features over a large range and the deficiency in extracting boundary information of high-level objects. This is attributed to the absence of feedback signals from higher-level visual cortices in the PHNet model. These signals participate in regulating the physiological activities of lower-level visual neurons to optimize information processing. Therefore, investigating the modulation of lower-level visual information by higher cortices may enhance the performance of biologically-inspired edge detection models in object boundary detection.

Overall, considering the extremely small parameter and computational cost of the PHNet model, its demonstrated edge detection performance is still satisfactory.

## Data availability statement

The datasets presented in this study can be found in online repositories. The names of the repository/repositories and accession number(s) can be found at: https://github.com/PXinTao/PHNet.

## Author contributions

XP was responsible for manuscript preparation and worked as a supervisor for all procedures. LZ and CL were responsible for programming and data processing. HY, YP, and YZ participated in discussions and revisions. All authors contributed to the article and approved the submitted version.

## Funding

The authors appreciate the anonymous reviewers for their helpful and constructive comments on an earlier draft of this paper. This work was supported by the National Natural Science Foundation of China (Grant No. 62266006), Guangxi Natural Science Foundation (Grant No. 2020GXNSFDA297006), National Natural Science Foundation of China (Grant No. 61866002), Project of the Key Laboratory of AI and Information Processing (Hechi University), Education Department of Guangxi Zhuang Autonomous Region (2022GXZDSY013), Guangxi Natural Science Foundation (Grant Nos. 2018GXNSFAA138122, 2015GXNSFAA139293), and 2022 Autonomous Research Project of Guangxi Key Laboratory of Automobile Components and Vehicle Technology (No. 2022GKLACVTZZ06).

## Conflict of interest

The authors declare that the research was conducted in the absence of any commercial or financial relationships that could be construed as a potential conflict of interest.

## Publisher’s note

All claims expressed in this article are solely those of the authors and do not necessarily represent those of their affiliated organizations, or those of the publisher, the editors and the reviewers. Any product that may be evaluated in this article, or claim that may be made by its manufacturer, is not guaranteed or endorsed by the publisher.
